# Extremely acidophilic filamentous fungi are more prevalent in diverse ecosystems than previously documented

**DOI:** 10.1038/s41598-025-06321-1

**Published:** 2025-08-19

**Authors:** Nguyen Thanh Thuy, Tom Coleman, Meera Christopher, Nguyen Bao Chau, Cao Xuan Bach, La Thi My Hanh, Efstratios Nikolaivits, Johan Larsbrink, Lisbeth Olsson, Vu Nguyen Thanh

**Affiliations:** 1 Food Industries Research Institute, 301 Nguyen Trai, Thanh Xuan, Hanoi, Vietnam; 2 Division of Industrial Biotechnology, Department of Life Sciences, Chalmers University of Technology, Gothenburg, Sweden; 3 Wallenberg Wood Science Center, Chalmers University of Technology, Gothenburg, Sweden; 4 Present Address: Industrial Biotechnology & Biocatalysis Group, School of Chemical Engineering, National Technical University of Athens, Athens, Greece

**Keywords:** New fungal taxa, Acidophile, Acidotolerant, Extremophile, Ascomycota, *Penicillium*, *Talaromyces*, *Acontium*, CAZymes, Biodiversity, Fungal biology, Fungal ecology, Hydrolases

## Abstract

The study of extremophiles can lead to the discovery of highly tolerant enzymes of value to biotechnology, and extreme environments are typically sampled to facilitate their discovery. Here, we show that extreme acidotolerant filamentous fungi, able to grow at pH < 1, can be found when sampling highly diverse environments, from industrial sites with low pH to typical non-acidic plant and soil samples. Over 100 fungal strains were isolated from over 2,000 samples taken from across Vietnam, and many of the strains were able to grow over wide pH ranges, displaying either acidotolerance or clear acidophilicity. ITS sequencing revealed that 63 isolates represent 12 previously undescribed species, with the majority from the *Talaromyces* or *Penicillium* genera. We furthermore report the rediscovery of the previously lost, historically significant acidophile *Acontium velatum*. Screening of selected fungal secretomes for polysaccharide-cleaving activity revealed that many show broad tolerance to harsh conditions (pH, temperature, organic solvents). Our work greatly expands on the diversity of identified extreme acidotolerant and acidophilic filamentous fungi, which can serve as sources of industrially relevant enzymes. For most species identified, acid tolerance or acidophilicity has not previously been reported, and our results showcase that acidophilicity is more widespread than previously appreciated.

**Supplementary Information:**

The online version contains supplementary material available at 10.1038/s41598-025-06321-1.

## Introduction

The existence of life in Earth’s extreme habitats, such as polar regions, deserts, volcanic areas, deep-sea trenches, and acidic streams, can give clues about the genesis of life, and inspire studies of evolution, biodiversity, and development of sustainable industrial processes^1,2^. However, the definitions of “extreme”, “-philic” and “-tolerant” can be unclear^3^. The most common proposed definitions for acidophilicity and acidotolerance, which will be used here, are that acidophiles grow optimally below pH 3.0, while acidotolerant strains grow optimally between pH 3.0 and 5.0^4,5^; furthermore, “extreme” organisms are those that can grow at pH 1. Notably, many filamentous fungi can be regarded as acidotolerant as their growth environments are commonly below neutral pH^6^, while strict acidophilic species are much less common. This can be attributed to the fact that highly acidic biospheres (pH < 3) are rare in nature^7^, and are predominantly a result of human activity.

Most research on extreme acidophilic and acidotolerant fungi has been associated with acid mine drainage sites, due to their environmental impact and unique biodiversity^8–10^. These extreme ecological systems are formed by continued exposure of sulfur-containing minerals (mainly pyrite) to atmospheric oxygen and moisture, allowing chemoautotrophic bacteria to convert sulfur to sulfuric acid, which dissolves and oxidizes iron to create hostile acidic ecosystems^10^. Despite these extreme conditions, the ecological diversity can be high and include known genera with acidotolerant and acidophilic fungi such as *Acidomyces*, *Aspergillus*, *Penicillium*^6,11^. Acidophilicity among fungi was first reported in 1943 with the identification of two species from acid solutions from industrial plants. These species were able to grow in a pH 1.0 medium^12^, and one was named *Acontium velatum*. The other species, amazingly, grew in a pH 0 medium containing 12% H_2_SO_4_ and was named “Fungus D”. Unfortunately, *A. velatum* appears to have been lost and is today unavailable in strain collections. In 1973, “Fungus D” was found to be conspecific to fungal strains from acidic soil near a natural gas purification plant and the novel species *Scytalidium acidophilum* (= *Acidomyces acidophilus*) was proposed^13^. Many organisms with extremotolerance to a single physical parameter (e.g. low pH) may be polyextremotolerant (tolerant to several parameters)^3^, suggesting that adaptation mechanisms may share common features. As industrial processes often take place under extreme pH and temperature or in the presence of harsh chemicals and solvents, studies on extremophilic species can be a useful way to identify industrially relevant organisms.

Past attempts to comprehensively list acidophilic fungi, using growth at pH < 4, predict the total known number of species to be low (81 total including 8 yeasts)^6^; by our count using stricter cutoffs, 15 taxa can be classified as acidophilic (Table S1). It is highly likely that more acidophilic species exist, but knowledge may be limited due to previous studies having focused on highly acidic environments, which are rare. Furthermore, many known species may have acidophilic traits but have yet to be assessed for these because they are from non-acidic environments^11^. We hypothesized that acidophilic species might be present in substrates such as soil and plant debris—possibly through the formation of highly localized, transient extreme microenvironments—and thus could be isolated from near-neutral environmental sites by using strongly acidic conditions as the selection criterion. To test this hypothesis, we chose to broadly survey the rich biodiversity of Vietnam^14–16^ for acidophilic filamentous fungi, from both acidic and non-acidic environments. From acidic environments, such as acid mine drainage sites (pH < 3), as well as typical soils and vegetation (pH > 3), totaling 2,240 samples, we were able to isolate 130 strains with either extreme acidophilic or acidotolerant phenotypes. Furthermore, the secretomes from a selection of these were screened for lignocellulolytic enzyme activities and tolerance to organic solvents. We report 12 undescribed species across six genera with potential value to biotechnology, and finally, we report the rediscovery of the previously lost and historic first-reported acidophilic filamentous fungus, *Acontium velatum*. We demonstrate here that the distribution of acidotolerant and acidophilic species is more widespread than previously shown, and that acidophilic species can be isolated from both highly acidic as well as close to pH-neutral environments.

## Materials and methods

### Fungal isolation using low pH as selection criterion

Acidotolerant and acidophilic fungi across various ecosystems were sampled from soil and plant debris from acidic regions (such as copper, pyrite, and coal mines, disrupted bogs, mangrove forests etc.) and unaffected rainforests, forestry plantations, rice fields, and gardens throughout Vietnam (Table S1, Supplemental File 1). The samples were stored in sterile polyethylene bags at ambient temperature before analysis. The samples were pulverized and sprinkled over acidified malt agar (2% malt agar containing 1% sulfuric acid, pH 0.8) plates, and incubated at room temperature for two weeks. Fungal growth was examined using a stereomicroscope (StereoBlue, Euromex). For purification and to confirm acid tolerance, hyphal tips were transferred onto a fresh isolation medium using a sterile tungsten needle. Successfully growing isolates were further cultured on malt agar for two weeks to facilitate sporogenesis and morphological differentiation through analyzing conidia structure, colony colors, textures, and sizes. Isolates with identical morphological appearance on the plates were grouped together, and one representative per group was selected to use for species identification through ITS sequencing. Since all isolates exhibited good growth in non-acidified media, standard malt agar medium was employed for routine cultivation and preservation. For long-term storage, both cryopreservation in liquid nitrogen and lyophilization were utilized.

### Determination of optimal growth pH

Optimal growth pH was evaluated using agar medium containing 1% sulfuric acid and 1% citric acid, which provides a strong buffer capacity from pH 0.96 to pH 6.50. To prepare plates with different pH values, a series of buffer solutions was prepared. For each buffer, 20 g of concentrated sulfuric acid and 20 g of citric acid were added to 50 mL of water. After complete dissolution, dry NaOH was added to adjust the pH to the desired values: pH 1.0, pH 1.5, pH 2.0, pH 2.5, pH 3.0, pH 3.5, pH 4.0, pH 4.5, pH 5.0, pH 5.5, pH 6.0, and pH 6.5. Each buffer solution was then brought to a final volume of 100 mL with water and autoclaved at 115 °C for 15 min.

For plating, 1 mL of each buffer solution was transferred into a Petri dish, followed by the addition of 19 mL of warm (50 °C) agar medium. The mixture was gently swirled to mix and allowed to solidify. Agar from each pH condition was subsequently collected, homogenized, centrifuged, and the pH of the liquid phase was measured and recorded as the actual pH values: 1.0, 1.6, 2.0, 2.5, 3.0, 3.5, 4.0, 4.5, 5.0, 5.6, 6.0, and 6.5 (final pH measurements are presented in Fig. 2). The difference between the initial and final pH values was likely caused by interactions between the medium components during autoclavation. The fungal isolates were grown on these plates for 2 weeks, after which they were photographed to evaluate growth.

### Gene barcoding and phylogenetic analyses

For phylogenetic analysis and species assignment, the Internally Transcribed Spacer (ITS) region was sequenced. DNA was extracted using phenol-chloroform (1:1, v/v) from a loopful of 48-hour old mycelia grown on malt agar. For dark pigmented isolates, extracted DNA was purified further using a Silica Bead DNA Gel Extraction kit (Thermo Scientific) to remove melanoid compounds that might interfere with PCR.

PCR amplification of the ITS regions was performed using any one of the previously reported forward primers ITS1 (5’ TCC GTA GGT GAA CCT GCG G 3’), ITS5 (5’ GGA AGT AAA AGT CGT AAC AAG G), ITS1F (CTT GGT CAT TTA GAG GAA GTA A 3’) or SR6R (5’ AAG WAA AAG TCG TAA CAA GG), and the reverse primer ITS4 (TCC TCC GCT TAT TGA TAT GC 3’). The amplicons were sequenced at First BASE Laboratories Sdn Bhd (Selangor, Malaysia).

ITS sequences of selected representatives from each genetic group have been deposited in GenBank (Table 1). The sequences were compared with available GenBank sequences using BLAST. For phylogenetic analysis, sequences were aligned using MAFFT (https://mafft.cbrc.jp/) running a progressive method with an accurate guide tree (G-INS-1)^17^. Phylogenetic trees were constructed using MEGA11^18^ and visualized using iTOL v8^19^.

### Production of secretomes

For evaluation of the potential of the fungi to produce and secrete lignocellulolytic enzymes, fungi were first grown on malt agar for one week at 30 °C, and the cell mass from one plate (prepared by cutting fungal colonies into agar blocks < 2 mm) then used to inoculate a 100 mL Erlenmeyer flask containing 1.5 g of malt residue (Weyermann malt), 1.5 g sugarcane bagasse (Lam Son sugar factory, Thanh Hoa, Vietnam), 1.5 g rice bran (from a local market, Hanoi, Vietnam), and 8 mL of mineral solution (4.0 g L^− 1^ KH_2_PO_4_; 13.6 g L^− 1^ (NH_4_)_2_SO_4_; 0.8 g L^− 1^ CaCl_2_·H_2_O; 0.6 g L^− 1^ MgSO_4_·7H_2_O; 1.0 g L^− 1^ yeast extract; 1.0 g L^− 1^ peptone; 0.2 mL L^− 1^ Tween 80; 1.0 mL L^− 1^ trace elements (3.2 mg L^− 1^ MnSO_4_·H_2_O; 2.8 mg L^− 1^ ZnSO_4_·7H_2_O; 10 mg L^− 1^ FeSO_4_·7H_2_O; 4.0 mg L^− 1^ CoCl_2_·6H_2_O; 3.5 mg L^− 1^ CuSO_4_·5H_2_O); adjusted to pH 2.5 with H_2_SO_4_). Solid state fermentation was carried out for 7 days at 30 °C. Secreted enzymes were extracted by addition of 45 mL 20 mM sodium citrate buffer, pH 5, agitation at 150 rpm for 1 h at 30 °C, and recovery of the crude enzyme solution by centrifugation at 4,000 rpm for 10 min at 10 °C. Secretomes were stored at -20 °C until use.

### Carbohydrate-active enzyme activity assays

Protein concentrations were determined using the Bradford assay. Xylanase and CMCase activities were measured using the 3,5-dinitrosalicylic acid (DNS) assay^20^. Briefly. 0.1 mL of appropriately diluted enzyme was mixed with either 0.2 mL of 1% (w/v) xylan from beechwood (Apollo Scientific Ltd.) or 1% (w/v) carboxymethylcellulose (CMC; Sigma-Aldrich), in a buffer at desired pH. The reactions were incubated at 50 °C for 20 min, and released reducing sugars quantified using the DNS assay, against standard curves of glucose or xylose. One unit per mL of enzyme was defined as the amount of enzyme releasing one micromole of reducing sugar per minute under the assay conditions. As a negative control, the enzyme extract from each strain was heated at 100 °C for 10 min, and all assay procedures were performed in the same way as for the test extract. The reducing sugar concentration from the control was then subtracted to account for substrate sugar carryover. To measure enzyme activities at different pH (pH 1, 3, 5, 7), Britton-Robinson buffer was used (40 mM each of boric, phosphoric, and acetic acids), titrated to the desired pH using 20% (w/v) of H_2_SO_4_ or NaOH. The same buffer was used to generate monosaccharide standard curves.

### Evaluation of thermal and solvent stability of enzymes

For thermal stability assays, the enzymes were diluted with 50 mM sodium citrate buffer, pH 5, incubated at 70–90 °C for 20 min, and then cooled at 4 °C overnight, followed by enzyme activity measurements at 50 °C and pH 5, as described above. Control samples prepared identically but without heating were used as controls. To test tolerance to organic solvents, the enzymes were diluted with 50 mM sodium citrate buffer, pH 5, containing organic solvent (acetone, acetonitrile or dimethylformamide at 10, 20, 30% v/v) or detergent (Sodium Dodecyl Sulfate (SDS), β-mercaptoethanol, TritonX-100, or Tween 80 at 0.1, 1 and 2% v/v), and enzyme activity measured as described previously. Standard curves were generated by diluting glucose and xylose with buffer containing solvent/detergent. PCA analysis was performed using the standard settings on OriginPro (OriginLab).

## Results

### Sampling in Vietnam and construction of a diverse library of filamentous fungi

**Fig. 1:**
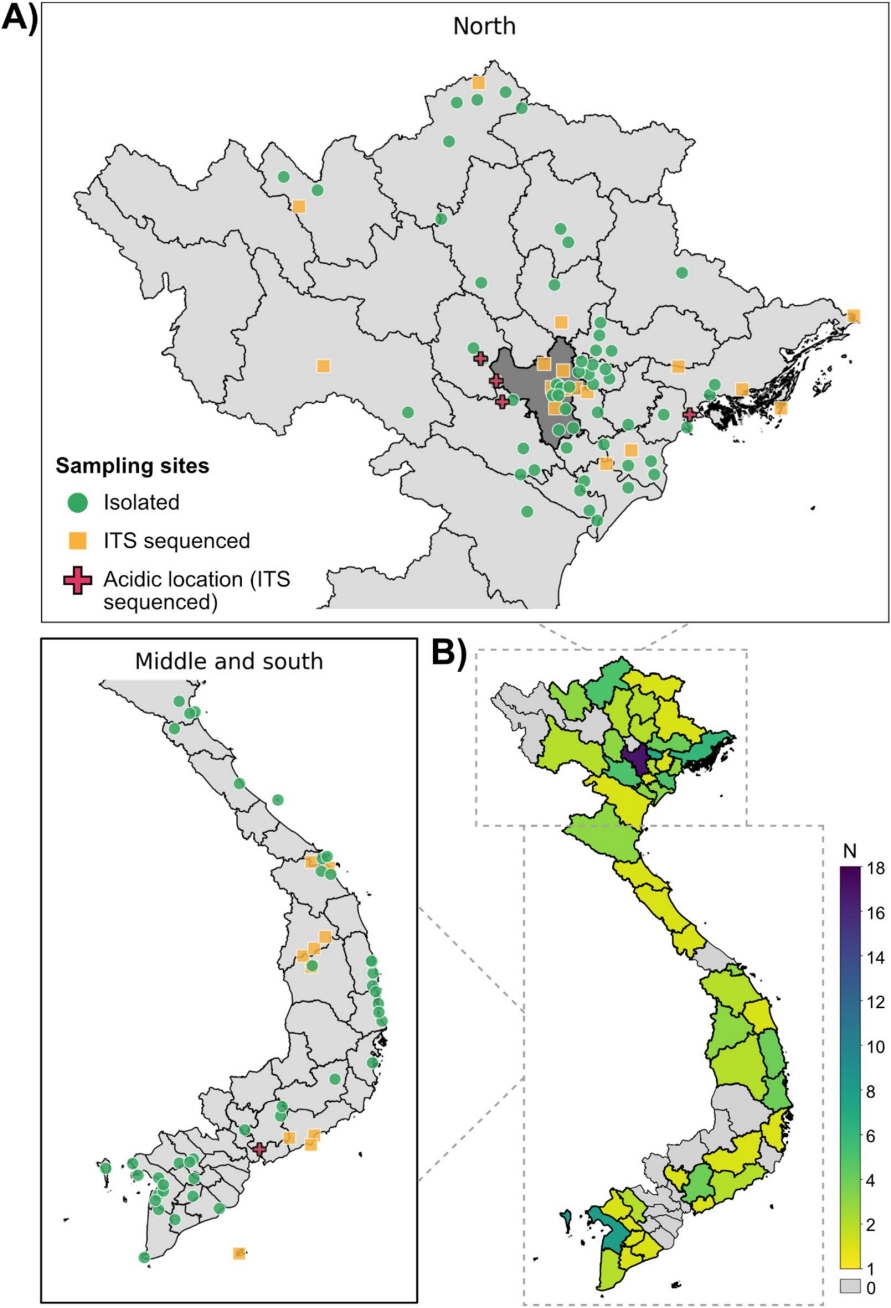
Origin of the acidotolerant and acidophilic fungal strains identified and screened. **(A)** Sampling locations. Green circles indicate coordinates of sampled locations where strains were subsequently isolated and determined to grow under acidic selection pressure. Yellow squares indicate the sampling sites where strains were selected for ITS sequencing. Red crosses indicate acidic environments where strains were successfully isolated under acidic conditions and ITS sequenced. Hanoi is shown in dark grey. Detailed sampling information, including coordinates, strain information and taxonomy, isolation location and environment are provided in Supplemental File 1. **(B)** Geographical heatmap of number of isolated strains (N) across Vietnam, sorted by province. Vector shapefiles were obtained from the Humanitarian Data Exchange (https://data.humdata.org/). The Python 3 code for this figure is provided in Supplemental File 2.

The microbial diversity in Vietnam is understudied, and thus provides an excellent opportunity for deepening our understanding of ecology in Southeast Asia. A selective solid medium containing 1% sulfuric acid (pH = 0.8) was employed for fungal isolation. Sulfuric acid provides strong buffering capacity at pH values below 2.5 (pKa values: -3 and 1.9), and is not likely to be assimilated in significant amounts by fungi. Between 2018 and 2024, 2,240 samples were collected from 131 sampling sites over a wide range of natural and anthropogenic ecosystems (Fig. 1, Supplemental File 1). Interestingly, extreme acidotolerant fungi were found in most of the sampling locations, although in low numbers. Directly placing samples on the low-pH agar plates typically yielded singular colonies after two weeks of incubation, while sporogenesis was rare (Figure S1). The plates were free of cosmopolitan species, such as *Fusarium*, *Trichoderma*, black aspergilli, and mucoraceous fungi. All the isolates grew well in standard non-acidified malt extract media, where sporogenesis was more intense, allowing for detailed morphological differentiation. From more than a thousand isolates, 130 that showed distinctive macromorphological characteristics were selected for species identification by ITS sequencing. Representatives from each species/genetic group were assessed for their growth pH optima, micromorphological characterization, and enzyme production on natural plant substrates.

### Taxonomy, ecology, and description of selected species

The sequenced ITS regions of the strains indicated that they all belong to Ascomycota, in agreement with previous compilations of known species of acidotolerant/acidophilic fungi^6^. Among the 130 strains, 67 could be identified as already known species, namely *Acidomyces acidothermus* (*N* = 23 strains), *Acrodontium griseum* (*N* = 20), *Penicillium citreonigrum* (*N* = 9), *Acontium velatum* (*N* = 6), *Acidomyces acidophilus* (*N* = 5), *Penicillium griseolum* (*N* = 2), *Talaromyces chlamydosporus* (*N* = 1), and *Aspergillus turcosus* (*N* = 1) (Table 1). The remaining 63 strains showed low homology in ITS sequences to known species and could be assigned to 12 as-yet-undescribed species, including four species in the genus *Talaromyces*, one species of *Phialomyces*, two species of *Acidomyces*, one species of *Penicillium*, one species of *Amplistroma*, and three species of unclear genetic status (Table 1). The ITS sequences obtained for representatives of all species and genetic groups have been deposited in GenBank with accession numbers PP646895-PP646950.

**Table 1:** Diversity of fungi isolated from plant residues and soil. The horizontal line separates novel species (above the line) with those previously identified.

Species	*N* strains	Acid affinity *	Selected strain **	GenBank number	Source of isolation	Location ***
*Acidomyces* sp1.	2	Acidophilic	ASA 2039.1	PP646903	Plant debris, mangrove forest	Phuoc An, Dong Nai
*Acidomyces* sp2.	6	Acidophilic	ASA 2062.4	PP646907	Plant debris, mangrove forest	Phuoc An, Dong Nai
*Amplistroma* sp1. aff. *A. erinaceum*	8	Acidophilic	ASS 88.1	PP646948	Forest soil	Kon Plong, Kon Tum
*Hypocreales incertae sedis* sp1.	1	Acidotolerant	ASS 64.1	PP646937	Hill soil	Luong Son, Hoa Binh
*Hypocreales incertae sedis* sp2.	8	Acidophilic	ASA 2052.1	PP646923	Plant debris, mangrove forest	Phuoc An, Dong Nai
*Hypocreales incertae sedis* sp3.	1	Acidophilic	ASA 1968.1	PP646924	Grasses, coal mine	Cam Pha, Quang Ninh
*Penicillium* sp1. aff. *P. dravuni*	4	Acidophilic	ASM 137.2	PP646912	Plant debris, acid waste	Duong Kinh, Hai Phong
*Phialomyces* sp1. aff. *P. macrosporus*	3	Tolerant	ASS 40.3	PP646919	Hill soil	Uong Bi, Quang Ninh
*Talaromyces* sp1. aff. *T. clemensii*	13	Acidophilic	AS 616.3	PP646927	*Luffa aegyptiaca* leaves	Dong Hung, Thai Binh
*Talaromyces* sp2. aff. *T. clemensii*	4	Tolerant	AS 607.6	PP646930	*Bauhinia variegate* flowers	Thanh Xuan, Hanoi
*Talaromyces* sp3. aff. *T. resinae*	12	Acidophilic	ASM 153.1	PP646935	Plant debris, pyrite mine	Ba Vi, Hanoi
*Talaromyces* sp4. aff. *T. iowaense*	1	n.d.	ASA 1792.1	PP646936	*Ficus racemose* rotten fruits	Mong Cai, Quang Ninh
*Acidomyces acidophilus* [MB#511856]	5	Acidophilic	ACA 17.1	PP646895	Acacia bark	Ba Vi, Hanoi
*Acidomyces acidothermus* [MB#804969]	23	Acidophilic	AST 152	PP646900	Plant debris, pyrite mine	Ba Vi, Hanoi
*Acontium velatum* [MB#142596]	6	Acidophilic	ASS 125.1	PP646940	Hilltop soil	Dong Van, Ha Giang
*Acrodontium griseum* [MB#308227]	20	Acidophilic	ASS 104.2	PP646945	Bazal soil	Pleiku, Gia Lai
*Aspergillus turcosus* [MB#506378]	1	Acidotolerant	ASS 350.1	PP646908	Cultivated soil	Me Linh, Hanoi
*Penicillium citreonigrum* [MB#165197]	9	Acidotolerant	AS 173.2	PP646909	Grass debris	Van Don, Quang Ninh
*Penicillium griseolum* [MB#302401]	2	Acidophilic	ASS 45.1	PP646915	Hilltop soil	Hoa Vang, Da Nang
*Talaromyces chlamydosporus* [MB#817392]	1	Acidophilic	ASS 52.1	PP646925	Garden soil	Loc Vuong, Nam Dinh

*Based on the criteria: Acidophile, optimal growth at pH 3.0 or below; acidotolerant, optimal growth at pH 3.0–5.0. “n.d.”, not determined.

**Strain ID in the collection maintained at FIRI, Hanoi, Vietnam.

***Coordinates for each location are provided in Supplemental File 1.

### *Acidomyces*

**Fig. 2:**
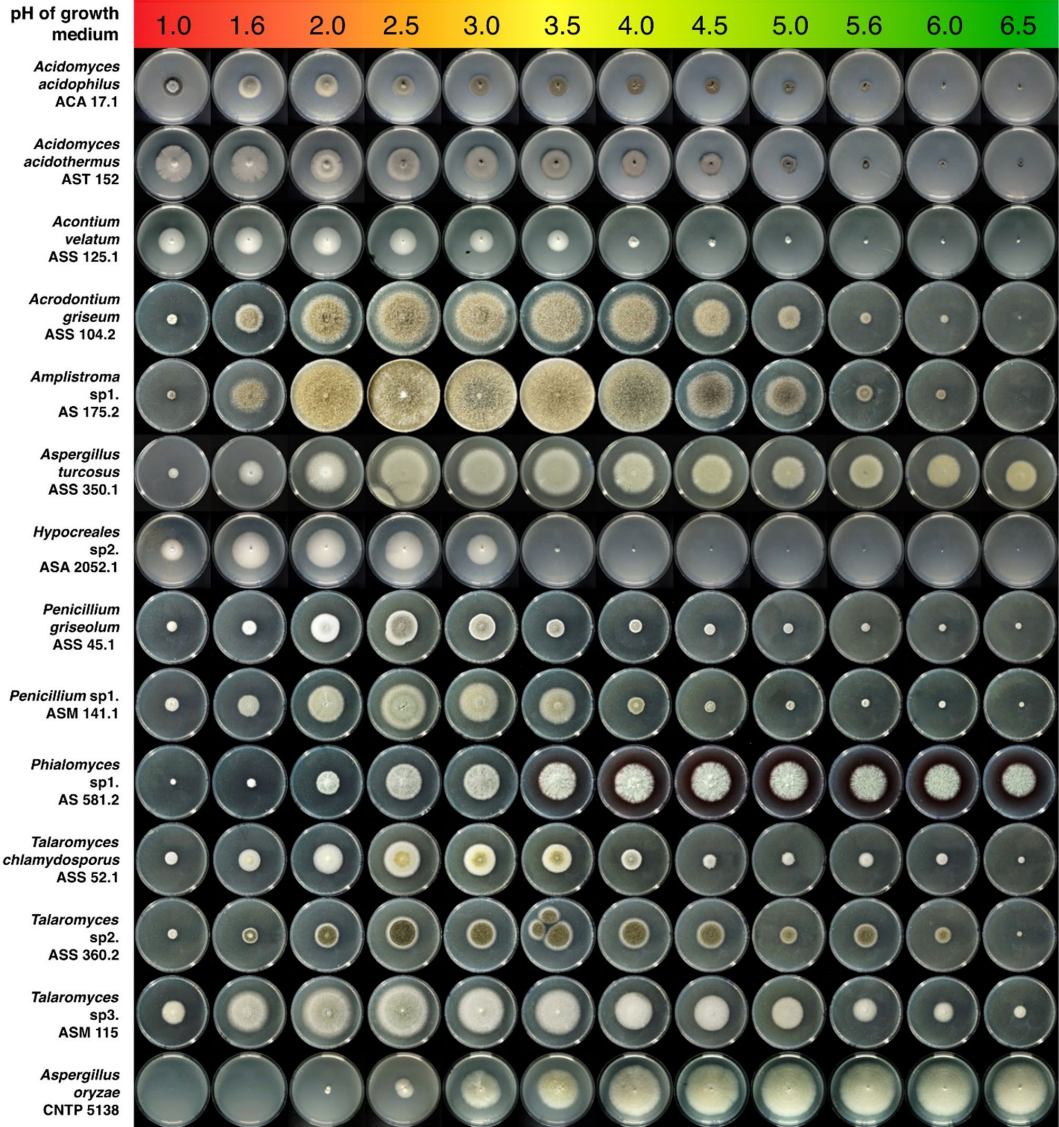
Growth of selected strains of acid tolerant/acidophilic fungi on buffered malt agar at different pH values. The media were buffered and contained 1% sulfuric acid and 1% citric acid. Agar plate pH values were checked at the end of the growth period to ensure there was no change. *Aspergillus oryzae* CNTP 5138 was used as a control strain, as an industrial, non-acidotolerant species. The fungi were grown at 28 °C for one week, except for the slow-growing *Acidomyces* spp. and *Acontium velatum* (two weeks).

With 36 isolates, *Acidomyces* was the most frequent fungal genus found in this study, and it was the most clearly dominant genus found in samples collected from extreme acidic environments (mine runoffs, disrupted mangrove forests, and factory acid waste streams). Previously, species of *Acidomyces* have been isolated from acid mine drainage biofilms, acid streams, sulfur contaminated soils, and acidic soils^13,21,22^. Significantly, we found *Acidomyces* strains also in non-acidic environments and from a vast variety of substrates, including soil, plant debris, bark, and leaves. All isolated *Acidomyces* strains exhibited optimum growth at pH < 3.0 and can therefore be considered true acidophiles according to our definition (Fig. 2).

**Fig. 3:**
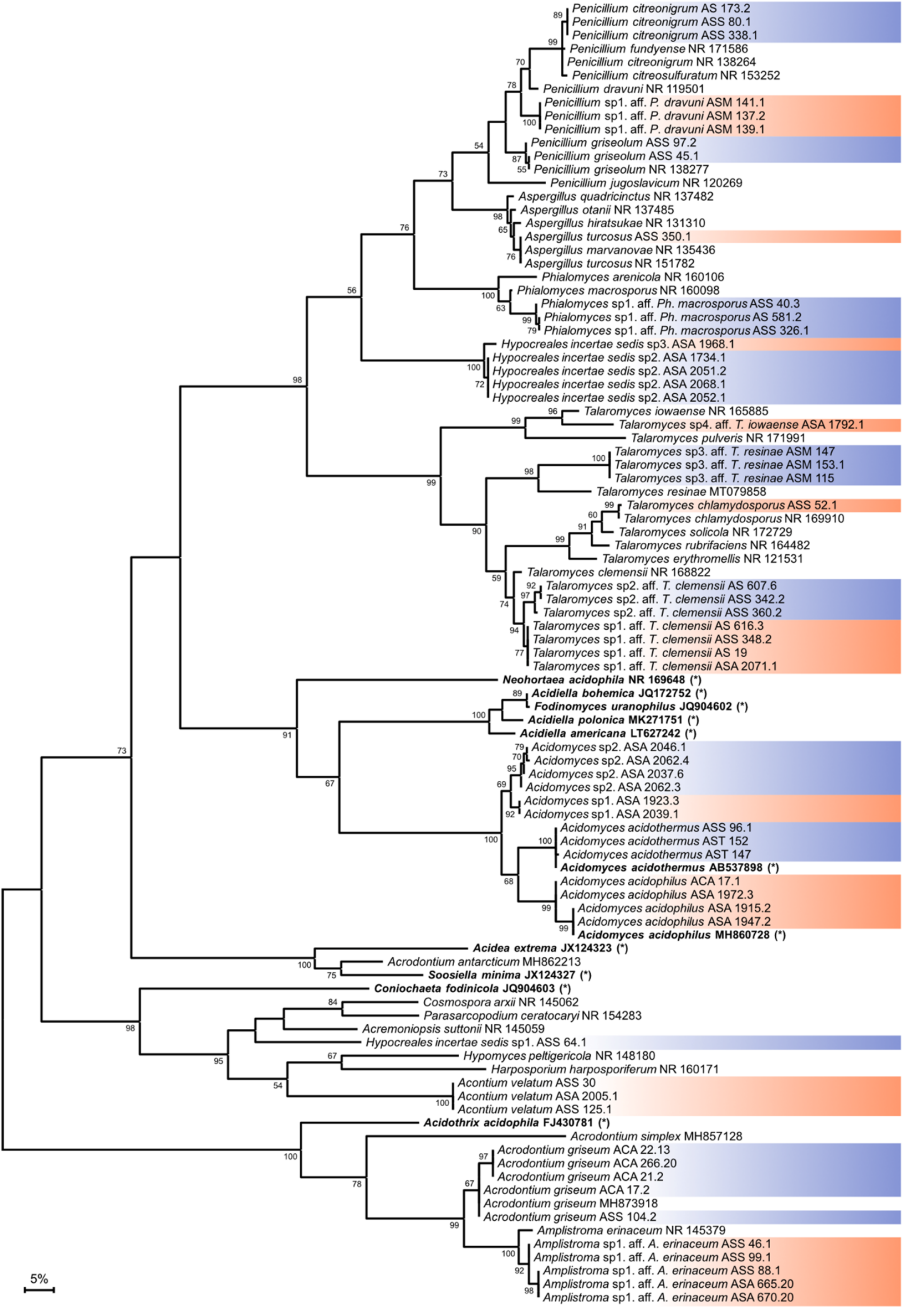
Maximum likelihood phylogenetic tree depicting the diversity of extreme acid tolerant/acidophilic fungi isolated from Vietnam, and their relationship to closely related/known extreme acid tolerant/acidophilic fungi. The tree was constructed based on ITS sequences using the Maximum Likelihood method and Tamura-Nei model in MEGA11^18^. An alignment of 870 positions was used for analysis. All ambiguous positions were removed for each sequence pair. GenBank accession numbers of ITS sequences are given after the taxon names. The scale bar represents 5% sequence divergence; genus groupings colored in alternating blue and orange (for clarity) represent strains obtained in this study; type strains are shown in black with no color bar; type strains are in bold and marked with an asterisk (*) are known extreme acid tolerant/acidophilic taxa.

To date, two species of *Acidomyces* have been described – *Acidomyces acidophilus* and *A. acidothermus* (= *A. richmondensis*)^23,24^. In our study, 23 strains were identified as *A. acidothermus*, and 5 strains as *A. acidophilus*. Phylogenetic analysis showed that a group of eight strains formed a well-supported cluster with *A. acidophilus* and *A. acidothermus* but represent two distinct lineages from those species (Fig. 3). The two lineages differed from each other by 2.4% in ITS sequence identity and from the known *Acidomyces* by 5.4% and 7.4%. The differences were significantly higher than the average weighted infraspecific ITS variability value of 1.96% for Ascomycota^25^. Here, we temporarily termed the groups *Acidomyces* sp1 (2 strains) and *Acidomyces* sp2 (6 strains). *Acidomyces* sp1 and *Acidomyces* sp2 formed grayish black, compact colonies with swollen chlamydospores and sterile aerial hyphae, typical for *Acidomyces* spp. The growth of *Acidomyces* sp2 was slow and more restricted compared to other *Acidomyces.*

With 23 strains isolated, *A. acidothermus* was the most frequent species found in this study and its name refers to its ability to grow in acidic conditions and at elevated temperatures (up to 45 °C)^22^. *A. acidophilus*, on the other hand, cannot grow at or above 37 °C^21^. Accordingly, we found *A. acidothermus* throughout Vietnam while *A. acidophilus* was restricted to Northern Vietnam, where the ambient temperature is significantly lower (Figure S2). The genome of *A. richmondensis* (= *A. acidothermus*) FRIK2901 has been sequenced, revealing a broad set of putative carbohydrate-active enzymes^26^. *Acidomyces* spp. are currently among the most studied acid tolerant/acidophilic fungi due to their unique physiology and technological potential^23^, and here it was evident that they are also widely distributed in diverse environments.

### *Acontium velatum*

**Fig. 4:**
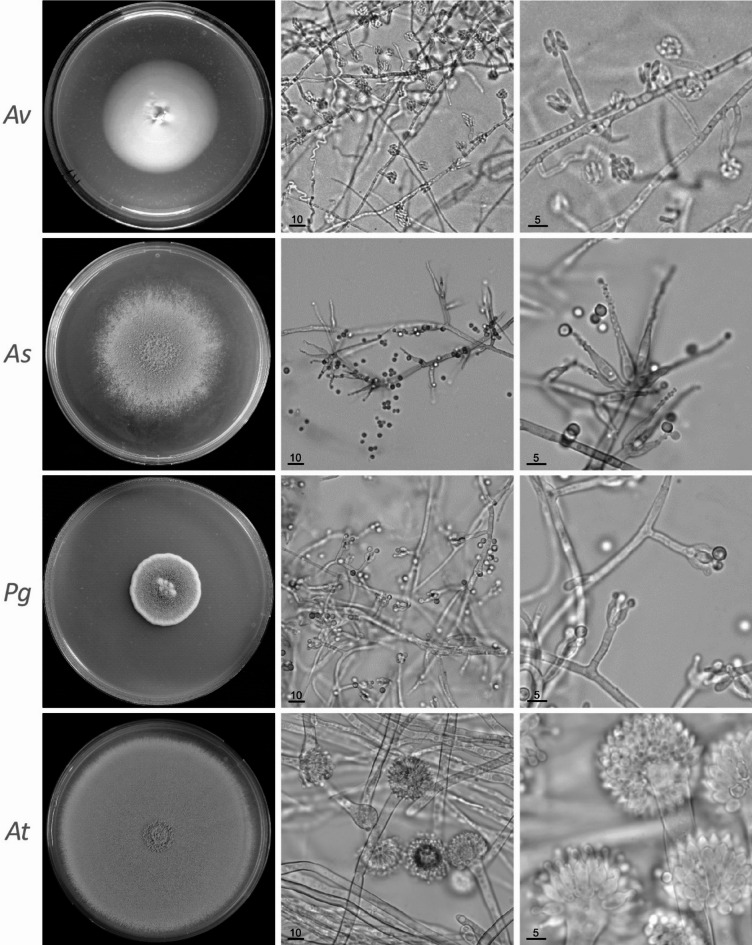
Plate cultures and conidiogenesis of selected acidophilic and acid tolerant fungi. By row: *Av* - *Acontium velatum* ASS 125.1 on malt agar containing 1% sulfuric acid, 3 weeks; *As* - *Amplistroma* sp1 ASS 88.1 on potato dextrose agar (PDA), 1 week; *Pg* - *Penicillium griseolum* ASS 45.1 on PDA, 1 week; *At* - *Aspergillus turcosus* ASS 350.1 on PDA, 1 week. Bars represent 10 μm (center) and 5 μm (right).

A group of six acidophilic fungi with similar morphology could not be affiliated to any known genera due to low ITS sequence similarity. These strains were associated with both acidic and non-acidic environments. Extreme acidophilicity of the fungus was suspected as it was found actively growing on wood samples stored in 1% sulfuric acid for almost 2 years. The strains produced white, spreading, yeast-like colonies on malt agar containing 1% sulfuric acid (Fig. 2). The observed growth optimum at pH < 2, combined with little to no growth at pH > 3.5, suggest *A. velatum* is a true acidophilic species. In old cultures, a thin, powdery white layer of mycelia with conidiophores was formed from the center of the colonies. The conidiophores were one-celled, elongated, and tipped with aggregated allantoid, bean-like conidia (Fig. 4). These characteristics fit well with the description of *Acontium velatum* mentioned in the first report on extreme acid tolerant fungi from 1943^12^, and thus the name was assigned to these strains (Fig. 3). Although *A. velatum* is one of the oldest cited examples regarding acidotolerance in fungi, the initial strain appears to have been lost and is not available in major fungal collections, but since the taxon *Acontium velatum* [MB#142596] is still legitimate, we here proposed the strain ASS 125.1, isolated from a cultivated rocky hilltop soil in Lung Tao, Dong Van District, Ha Giang, Vietnam (coordinates 23°17’35.2"N 105°18’16.3"E) in 2019, as the neotype for the species. The strain is now maintained in a metabolically inactive state at the Vietnam Collection of Industrial Microorganisms, Food Industries Research Institute, Hanoi, Vietnam. The ITS sequence for the strain has been deposited in GenBank with ID: PP646940.

### *Acrodontium* and *Amplistroma*

A group of 28 strains sharing similar morphology and distribution formed a well-supported cluster in the ITS sequence tree with *Acrodontium griseum* and *Amplistroma erinaceum*; neither genus has previously been associated with acid tolerance (Fig. 3). The strains were found in soil and plant debris throughout Vietnam and there was no clear preference for acidic environments. On the isolation plates, the light to dark brown fungi showed a moderate level of conidiogenesis. They formed one-celled apiculate conidia borne on sympodial straight and denticulate conidiogenous cells. Twenty isolates were identified as *A. griseum* based on ITS sequences. The species has been listed among fungi found in compost^27^, endophytes in orchid (*Aularthron bilamellatum*)^28^ and creeping thistle (*Cirsium arvense*)^29^. The remaining eight isolates were closely related to *A. erinaceum* but differed from the latter by 15/594 nucleotides. Classification of *Amplistroma* is mainly based on morphology of fruiting bodies formed on native plant substrates. Among nine validly described species of *Amplistroma*, four have not been cultured^30^, making it difficult to draw conclusions on the species’ behavior in acidic conditions.

### *Aspergillus turcosus*

Despite the cosmopolitan nature and genetic diversity of *Aspergillus*, in this study, only one strain of acid tolerant *Aspergillus* was found, and it was identified as *Aspergillus turcosus* (Figs. 2 and 4). Strains of *A. turcosus* have been isolated previously from clinical samples and mistakenly treated as *A. fumigatus*, and the primary reason for interest in *A. turcosus* is thus related to its clinical importance and azole drug resistance^31^. The genome of *A. turcosus* encodes a broad carbohydrate-active enzyme repertoire typically found in saprotrophic Aspergilli^32^. The lack of diverse *Aspergillus* strains in our study is noteworthy as several species have been previously linked to acidotolerance^6^.

### *Penicillium* and *Talaromyces*

A group of 46 morphologically heterogenous isolates were identified as species of *Penicillium* and *Talaromyces* (Fig. 3), but, strikingly, no correlation to acidic sampling environments was observed. On primary isolation plates, the fungi showed different adaptation to extreme acidity indicated by the growth patterns and levels of conidiogenesis. *Penicillium* and *Talaromyces* represent diverse groups of fungi with worldwide distribution, and furthermore play an important role as decomposers of organic matter^33,34^. The genus *Talaromyces* was introduced to accommodate a group of *Penicillium* in the *Biverticillium* that possess sexual stages^35^. Currently, the genus *Penicillium* contains 483 species and the genus *Talaromyces* 171 species^36^. Strains of both *Penicillium* and *Talaromyces* have been previously isolated at pH 3, at times showing optimal growth at pH values as low as 2^37,38^, which clearly indicates acidophilicity.

The ITS sequences of nine isolates were closest to *Penicillium citreonigrum*, which has previously been genome sequenced (https://mycocosm.jgi.doe.gov/Pencit1)^39^, but for which no information on acidophilicity or tolerance has been reported. The assignment of the isolates to *P. citreonigrum* is however provisional as there are several closely related species in this group, including *P. fundyense* and *P. citreosulfuratum.* Two isolates were identified as *P. griseolum*, which was originally isolated from acidic dune sand in England. The fungus formed monoverticillate, short and smooth conidiophores with globose, grey conidia. The morphology of *P. griseolum* is unique among *Penicillium* and the species represent a monophyletic section within the genus^36^. The species is a frequent endophyte in the root of conifers *Pinus monticola*, *Pinus ponderosa*, and *Pseudotsuga menziesii*^40^. Similar to *P. citreonigrum*, the tolerance of *P. griseolum* to extreme acidic conditions has not yet been reported. Four isolates were close to *Penicillium dravuni* but differed from the later by seven nucleotides. The species was described in 2005, based on a strain isolated from marine alga *Dictyosphaeria versluyii*^41^.

Among 31 strains assigned to the genus *Talaromyces*, only one strain could be identified on the species level based on its ITS sequence, as *Talaromyces chlamydosporus*, but since this species was described only recently^42^, predictions or assumptions regarding behavior in acidic conditions cannot be made. The remaining strains represent four lineages, including two closest to *Talaromyces clemensii*, one to *T. resinae*, and one to *T. iowaense*. The differences in ITS sequences to related species were high, excluding the possibility of species-level assignment.

### *Phialomyces* and *Hypocreales incertae sedis*

A group of three grayish-olivaceous pigmented strains formed a cluster with *Phialomyces arenicola* and *Ph. macrosporus* but represent a distinct lineage (Fig. 3). It differed from the closest species *Ph. macrosporus* by 14/510 nucleotides. The strains grew over a wide pH range, from 2.5 to 6.5, and optimal growth between pH 3.5–4.5 and thus were deemed acidotolerant (Fig. 2).

Ten strains forming two deep-rooted lineages could not be assigned to known genera based on ITS sequences, and hereby are regarded as *Hypocreales incertae sedis* (Table 1). These strains were slow-growing and produced conidia in chains on the tip of simple single cell phialides. One slightly pinkish strain represents a previously unidentified species, *Hypocreales* sp1, which was acidotolerant. Meanwhile, the remaining nine strains were non-pigmented, representing two unidentified species here called *Hypocreales* sp2 and *Hypocreales* sp3 which were both acidophilic. Their growth was remarkably restricted to acidic conditions with pH below 3.5 and optimum at pH 2.0 (Fig. 2) and we thus consider them extreme fungal acidophiles worthy of future deeper investigations.

### Enzyme screening and characterization

#### Screening of secreted carbohydrate-active enzyme profiles

**Fig. 5:**
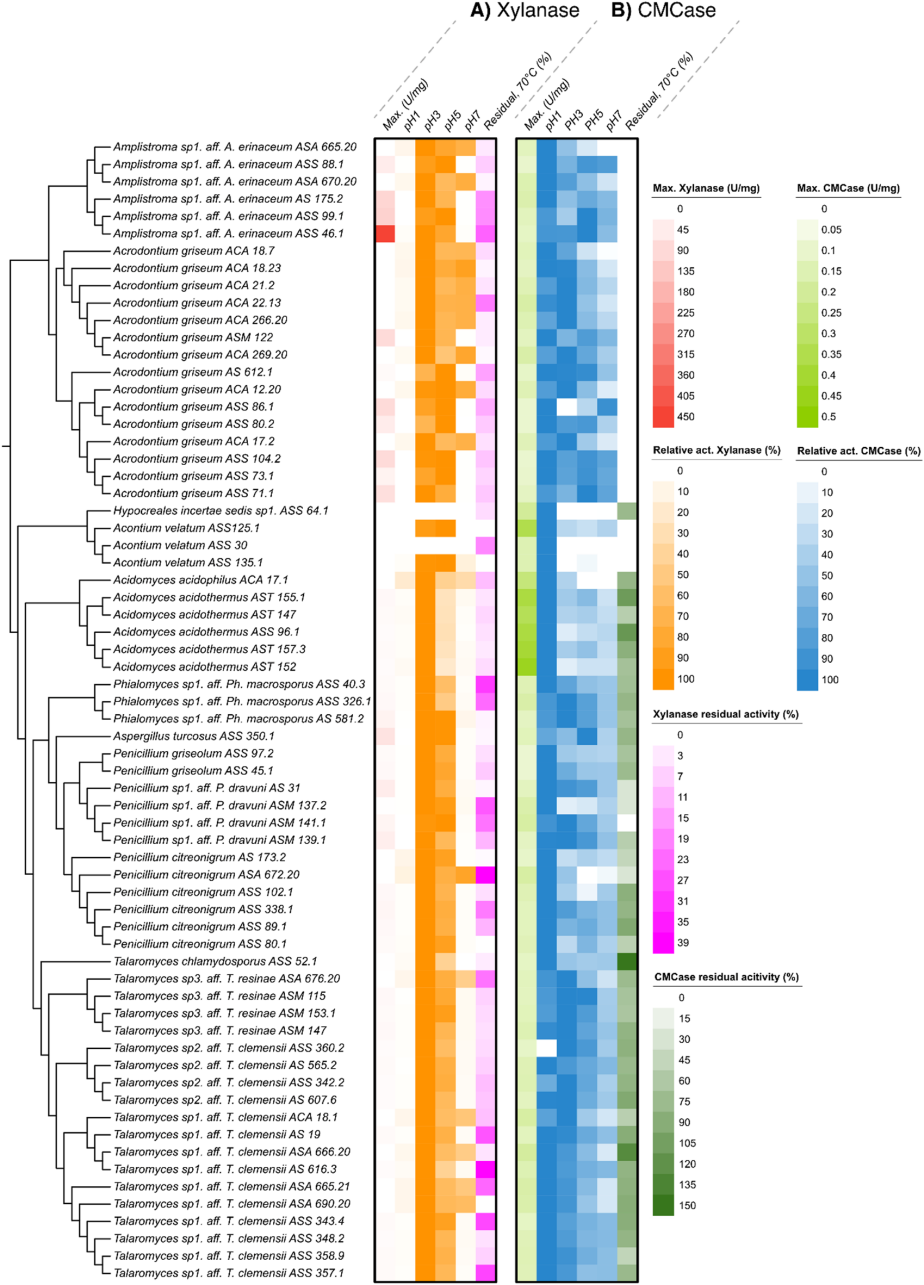
Measurement of (A) xylanase and (B) CMCase activities of the secretomes of acidotolerant and acidophilic fungi grown on complex substrates (1.5 g/100 mL each of malt residue, sugarcane bagasse, and rice bran). All activities were measured at 50 °C. In each panel is shown the maximum specific activity (U/mg); the relative activity level at pH 1, 3, 5 and 7, scaled to the highest activity observed as 100%; and lastly the residual activity after heating at 70 °C for 20 min followed by overnight cooling at 4 °C before measuring activity at 50 °C. Each value is the average of duplicate measurements. Blanks containing no substrate were subtracted. On the left is shown an unscaled UPGMA (unweighted pair group method with arithmetic mean) tree.

Filamentous fungi are known for secreting a diverse array of enzymes when growing on complex substrates. In our previous report on acidotolerant thermophilic lignocellulolytic fungi, many strains were found to have higher carboxymethylcellulose hydrolytic activity (CMCase) at pH 3 than at pH 5^16^. Given the high acid tolerance during growth observed in the species studied here, we speculated that also their enzymes may have such properties, even when sourced from non-acidic environments. Thus, assessing the pH optimum of secretomes may reveal trends between genera. Several strains of each newly discovered species (where possible) were cultivated on a complex medium containing both sugarcane bagasse and rice bran to potentially induce broad lignocellulolytic activities. The proteins secreted into the media (secretomes) were collected and concentrated, followed by xylanase and CMCase assays (which are representative assays for lignocellulolytic enzyme activities) conducted at pH 1, 3, 5 and 7 (Fig. 5). Xylanase activity was typically highest at pH 3, with drastic reduction in activity both above and below this value (Fig. 5A). In contrast, the CMCase activity was broader for most of the secretomes and typically maximal at pH 1 (Fig. 5B). We here performed controls to rule out the possibility of non-enzymatic acid hydrolysis, as hydrolysis is faster at low pH. This check showed that significant acid hydrolysis occurred only at pH 1, which was subtracted from the appropriate assay results, and thus acid hydrolysis was not responsible for the observed activity. The highest xylanase activity (451 U/mg protein) was observed with the secretome of *Amplistroma sp1.* (strain ID: ASS 46.1), with a maximum at pH 3. For CMCase activity, the highest activity was seen for the secretome from isolate AST 152 of *Acidomyces acidothermus* which was most active at pH 1 (0.44 U/mg). While the absolute value of CMCase activity was low, and it being an unnatural substrate for general endo-glucanase activity screening, these results indicate that several secretome enzymes have optimal activity at extremely low pH.

#### Tolerance to organic solvents, surfactants and heat

Our discovery of multiple fungi able to grow in acidic conditions—from both known and hitherto unknown species—and their production of apparently acid-preferring enzymes, inspired us to assess enzyme tolerance to other conditions^43^. Assays were performed as previously described, but additionally included common organic solvents and surfactants: acetone, acetonitrile, dimethylformamide (DMF; each at 10, 20, 30% v/v), sodium dodecyl sulfate (SDS), β-mercaptoethanol (BME), Triton X and Tween 80 (each at 0.1, 1, 2% v/v), and compared to control reactions without added organic solvents or surfactants.

The xylanase activity was typically reduced to < 50% of initial activity after inclusion of organic solvents or surfactants in the assay, and we therefore consider a particular species’ secreted enzymes “resistant” to an organic solvent or surfactant if the residual activity was ≥ 50% of the control reaction. The results showed that several strains of *Aspergillus*, *Penicillium*, *Phialomyces* and *Talaromyces* had secretomes with xylanases resistant to certain organic solvents and surfactants (Figure S3). One notable example was the strain *Amplistroma* sp1. aff. *A. erinaceum* (ASS 99 − 1), which retained ≥ 50% residual activity in the presence of 20% v/v acetone, 1% BME, 1% Triton X, or 1% Tween 80.

Principle component analysis (PCA) was used to investigate similarities and differences on how each solvent influenced the xylanase activity. Two eigenvectors explained 82.7% of the variance (PC1, 66.3%; PC2, 16.5%, Figure S4), and the loading coefficients were < 0.5 for all chemicals tested (including the control condition) on PC1 (Table S2), suggesting that each chemical affected the xylanase activity in a similar fashion. When analyzing CMCase activity, in contrast to the xylanase results, the addition of acetone, acetonitrile, DMF, as well as the anionic SDS increased the observed activity significantly (Supplemental File 3). It cannot be excluded, however, that these observed activity increases stem from an interaction with the reducing sugar assay used to monitor activity.

We next assessed the thermotolerance of each species’ secreted enzymes by heat-treating each secretome at 70 °C before analysis of residual enzyme activity. No significant thermotolerance was observed for xylanase activity, where the residual activity after heat treatment was < 30% compared to the untreated samples for the majority of secretomes (Fig. 5A). However, the secretomes from several *Acidomyces*,* Aspergillus*,* Penicillium*, and *Talaromyces* strains retained over 50% and up to 100% of CMCase activity after heat treatment (Fig. 5B). Thermotolerance has been reported for exo- and endo-glucanase activity for some strains previously^44^. The *Acrodontium* and *Amplistroma* secretomes on the other hand had < 25% residual activity in all cases, which points towards production of much less thermotolerant enzymes. When compared to well-known industrially relevant filamentous fungi, namely *Talaromyces cellulolyticus* and *Aspergillus niger*, the enzymes secreted by many members of our collection of fungi displayed similar tolerance to very low pH, as well as elevated temperature. Previously, β-glucosidases from *T. cellulolyticus* have been shown to have maximum activity at pH 5.5–6, while the CMCase activity of its cellulases was optimal at pH 4^45^. While *A. niger* can grow at a remarkable range of pH values (1.4–9.8), the pH optimum of its cellulases have been typically reported as pH 4.0, with optimal stability at pH 5.0 and above^46–48^. Selected members of our acidophile culture collection can be considered competitive with, or more tolerant to, these desirable properties. In summary, the activity results highlight that enzymes from many fungal genera—including several previously not linked to acidotolerance—possess high tolerance to multiple physical conditions, and this phenomenon may be relatively common.

## Discussion

Fungal biodiversity is high in tropical regions, and through application of selection pressures, can be effectively filtered to identify new species with targeted properties. We here used growth at low pH as a moderate selection pressure and were able to expand greatly on the known diversity of acidotolerant and extreme acidophilic fungi. We found a surprisingly high diversity of strains able to grow under this selective pressure even outside of locations considered acidic. We can here report that in previously classified taxa, six additional species can be added to the list of acidotolerant and/or acidophilic filamentous fungi, which adds to the functional diversity of known species. Additionally, we provide genetic evidence that the newly isolated strain ASS 125.1 represents a candidate for the neotype of the previously lost acidophile, *Acontium velatum*. Furthermore, 12 taxa were identified as being entirely new, the majority of which growing preferentially at low pH, from known genera with acidophilic species, genera previously not linked to acidophilicity, as well as potentially new and previously undescribed genera.

Highly acidic biospheres (pH < 3) are rather rare in nature^7^, and our observation of widely spread acidotolerant and acidophilic fungi from acidic to non-acidic environments prompts speculation about the true ecological habitats of these fungi. Except for volcanic springs, most highly acidic environments found today are of anthropogenic origins, and have not exerted selective pressure for millennia. The most prevalent natural acidic environments might instead be associated with acid-containing plant tissues, which especially saprophytic or plant-pathogenic fungi would often encounter. Although most organic acids are weak, some commonly found in plant tissues such as oxalic acid may cause extreme acidification, especially when the tissue is withering up and the acid consequently becomes concentrated^49,50^. For example, 10 mM and 100 mM solutions of oxalic acid have pH values of 2.09 and 1.31, respectively. Furthermore, there is a wide range of fungi that can produce oxalic acid both when grown on laboratory media and on native substrates^51^. Oxalic acid plays several biological roles, including initiating the necrosis of plant tissue during pathogenesis, for competition with other species, for scavenging trace minerals, and mobilizing phosphate and sulfate ions^52^. Thus, extreme acid tolerance might not necessarily be rooted from adaptation to acidic geo-habitats but could be the result of fungal-plant interactions. The biology of these fungi is poorly understood, and these newly isolated species provide a great opportunity for further study.

In the current energy industry, there is a growing need for the use of lignocellulose residues as renewable carbon feedstocks to replace fossil resources^53^, particularly wheat straw in Europe^54^ and rice straw in Vietnam and across Southeast Asia^55,56^. The library of species identified here was observed to secrete CAZymes when grown on lignocellulose, and many with optimal activity at very low pH. Potentially these fungi can provide two advantages in lignocellulose deconstruction applications – first, by operating in acidic conditions, where the competition from mesophilic organisms would be reduced; and second, the deconstruction of biomass may be accelerated due to the acidity. Second-generation biofuel production often utilizes steam explosion or other pretreatment methodologies that lead to acidification of the feedstock^57^. For continuous bioprocessing, enzymes and microorganisms should retain activity at the pH of the side-stream, as well as tolerate inhibitors present. By discovering and applying enzymes adapted for such conditions, costs connected to both neutralization and microbial contamination—which are common in conditions closer to neutral pH—could potentially be mitigated.

Significantly, many of the here studied secretomes were found to have tolerance to organic solvents and surfactants, in addition to low pH, and these may be useful traits in industrial settings. For CMCase activity, there was an added tolerance also to elevated temperatures, suggesting overall robust enzymes secreted by many of the fungal strains, which has also been observed in previous studies on novel *Thielavia* spp. strains exhibiting both thermal stability and tolerance to acidic conditions^16^. In contrast, however, there was no cross-tolerance towards elevated temperatures for xylanase activity in our experimental setup, which may reflect on the sampling strategy not selecting for thermotolerant strains. Future in-depth biochemical characterization of selected enzymes from the here discovered fungi would be needed to conclusively demonstrate enzymatic properties and industrial relevance.

In conclusion, multiple new acidotolerant and acidophilic strains were isolated using low pH as the main selection criterion, and it was shown to be a successful strategy to sample functional diversity across the whole country of Vietnam from both acidic and non-acidic environments. We suggest that the design of such selection pressures should be a key part of larger environmental sampling campaigns, to assay different phenotypes or functional range within different biotopes. We speculate that acidophilic strains are much more prevalent than is recognized today, and that such phenotypes have been neglected in previous work. Our results suggest that an expectation that the pH dependence of a species is closely tied to the surrounding environment is not necessarily true, and that organisms exhibiting extreme acidophilic traits can exist in habitats without apparent highly acidic character.

The 130 newly isolated strains are now maintained in the Vietnam Collection of Industrial Microorganisms at the Food Industries Research Institute which are available for academic use, and which represent a remarkable level of diversity. Further investigation of these remarkable new species, through genome sequencing and annotation, will be highly relevant for both fundamental biological study of acid-tolerance, as well as to pinpoint which enzymes they employ for successful depolymerization of complex renewable plant biomass at extremely acidic conditions.

## Electronic supplementary material

Below is the link to the electronic supplementary material.

Supplementary Material 1

Supplementary Material 2

Supplementary Material 3

Supplementary Material 4

Supplementary Material 5

## Data Availability

All data are contained within the manuscript or associated online supplemental information. Raw data used for calculations are available on reasonable request from the corresponding author. The ITS sequences obtained for representatives of all species and genetic groups have been deposited in GenBank with accession numbers from PP646895 to PP646950.

## Abbreviations

CAZyme: Carbohydrate active enzyme
Extreme acidophilic: Species which grows at pH 1.0 and optimally at pH 3.0 or below
Extreme acid-tolerant: Species which grows at pH 1.0 and optimally between pH 3.0 and 5.0
SDS: Sodium dodecyl sulfate
CMC: Carboxymethyl cellulose

## Declarations

### Adherence to national and international regulations

All work described in this manuscript has been performed under a memorandum of understanding between FIRI and Chalmers University of Technology.
