# Supplementary material for: Extremely acidophilic filamentous fungi are more prevalent in diverse ecosystems than previously documented
Source: Sci Rep. 2025 Aug 19;15:30445. doi: 10.1038/s41598-025-06321-1 (PMC12365195; doi:10.1038/s41598-025-06321-1)
Supplement: Supplementary file 2 — Supplementary Material 2 [file 41598_2025_6321_MOESM2_ESM.pdf]

Supplementary Information for:

Extremely acidophilic filamentous fungi are more prevalent in diverse ecosystems than previously documented

Nguyen Thanh Thuy[1,*], Tom Coleman[2,*], Meera Christopher[2,3], Nguyen Bao Chau[1], Cao Xuan Bach[1], La Thi My Hanh[1], Efstratios Nikolaivits[2,†], Johan Larsbrink[2,3], Lisbeth Olsson[2,3], Vu Nguyen Thanh[1,‡]

[1] Department of Microbiology, Food Industries Research Institute, 301-Nguyen Trai, Thanh Xuan, Hanoi, Vietnam

[2] Division of Industrial Biotechnology, Department of Life Sciences, Chalmers University of Technology, Gothenburg, Sweden

[3] Wallenberg Wood Science Center, Chalmers University of Technology, Gothenburg, Sweden

* These authors contributed equally to this work.

[†] Present address: Industrial Biotechnology & Biocatalysis Group, School of Chemical Engineering, National Technical University of Athens, Greece

[‡] Corresponding author, thanhvn@firi.vn

Table of contents

Figures and tables

Figure S1. Isolation of acidophilic fungal strains.

Figure S2. Distribution of identified species.

Figure S3. Resistance of fungal secretomes to organic solvents and surfactants.

Figure S4. Principle Component Analysis of organic solvents and surfactants.

Table S1. Current taxonomic positions of acidophilic and acid tolerant fungi, and relevant references.

Table S2. PCA loading coefficients for each organic solvent and surfactant.

Additional Supplemental Files

Supplemental File 1. Excel spreadsheet containing all sampling sites (province and district), strain sampling and geospatial data, and identifications for all species found during the study.

Supplemental File 2. Python3 code used to generate Figure 1.

Supplemental File 3. Full activity data for secretomes of selected strains when tested with organic solvents and surfactants. For each species is shown: the secretome protein concentration; xylanase and CMCase specific and residual activity (compared to 0% solvent), when assayed with several concentrations of each solvent or surfactant; relative activity levels at pH 1, 3, 5, and 7. These data were used to generate Figure S3 and 4.

Supplemental File 4. Full xylanase and CMCase activity data for secretomes of selected strains, containing a range of tested pH and temperature values. For each species is shown: the secretome protein concentration; xylanase activity at different pH (both specific activity and normalized to maximum activity); CMCase activity at different pH; tolerance of secretome enzyme activity when incubated at 70°C (both xylanase and CMCase) and compared to conditions where no elevated temperature was applied. These data were used to generate Figure 5.

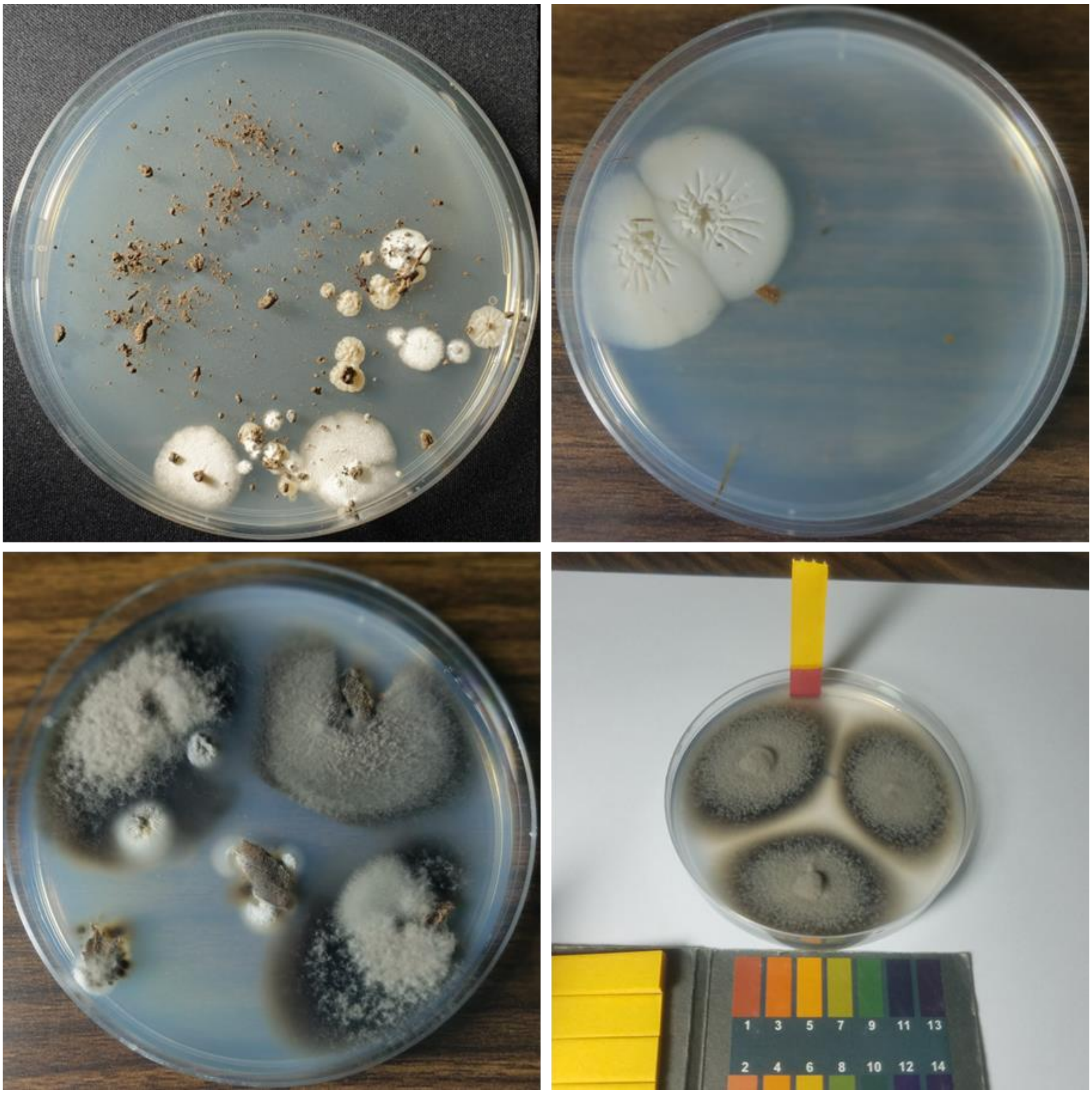

Figure S1. Isolation of acidophilic fungal strains. Samples (soil, plant residues) were sprinkled over 2% w/v malt agar plates containing 1% v/v sulfuric acid and incubated at 28 °C for 3 weeks. The above photos show typical fungal growth on the isolation plates. The litmus paper strip demonstrates the measured pH of the medium.

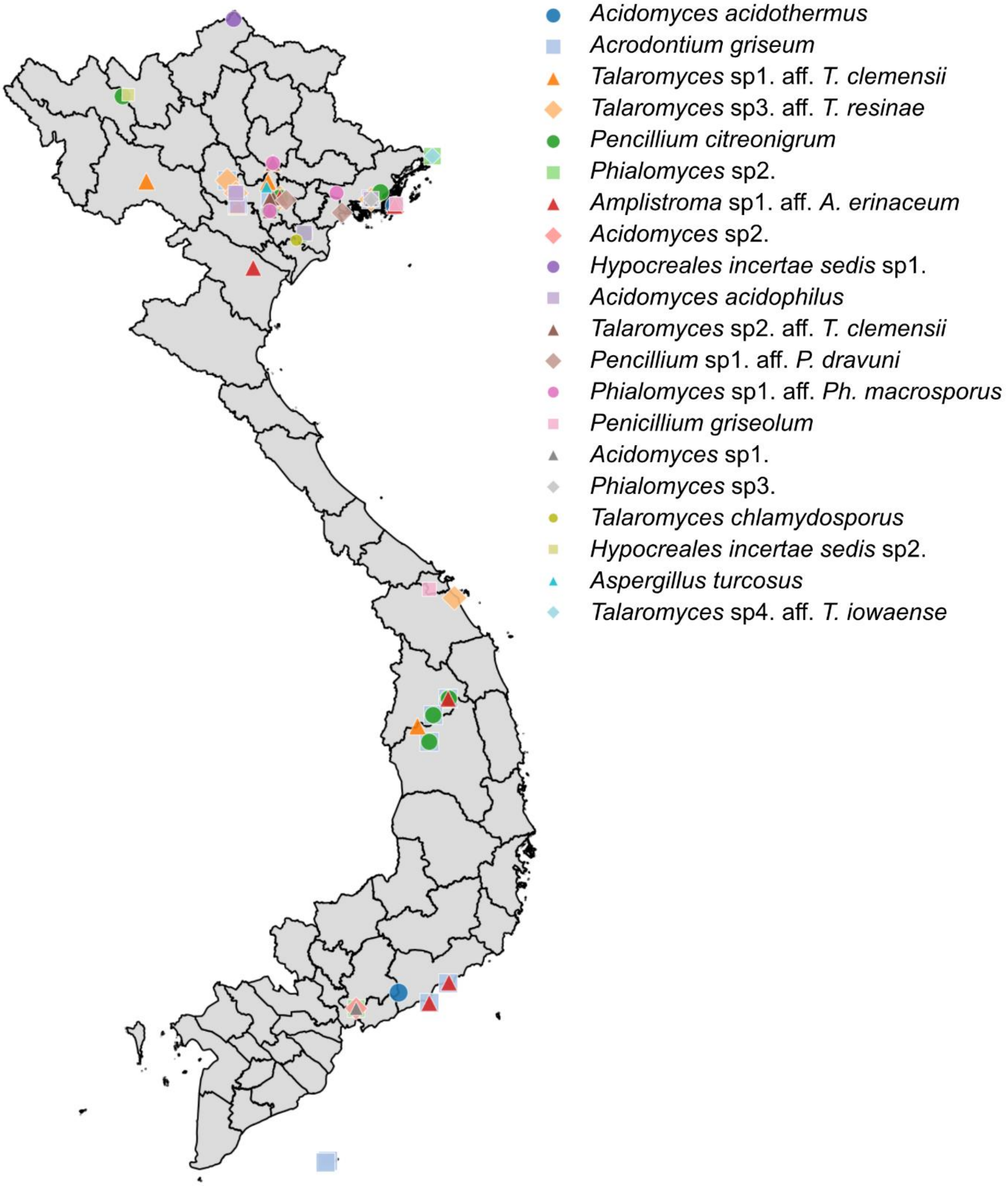

Figure S2. Distribution of identified species. The species list is in descending order of number of strains isolated, and the marker sizes are scaled slightly for more prevalent species. The exact coordinates are provided in Supplemental File 1. Vector shapefiles were obtained from the Humanitarian Data Exchange (https://data.humdata.org/) under a CC BY-IGO license.

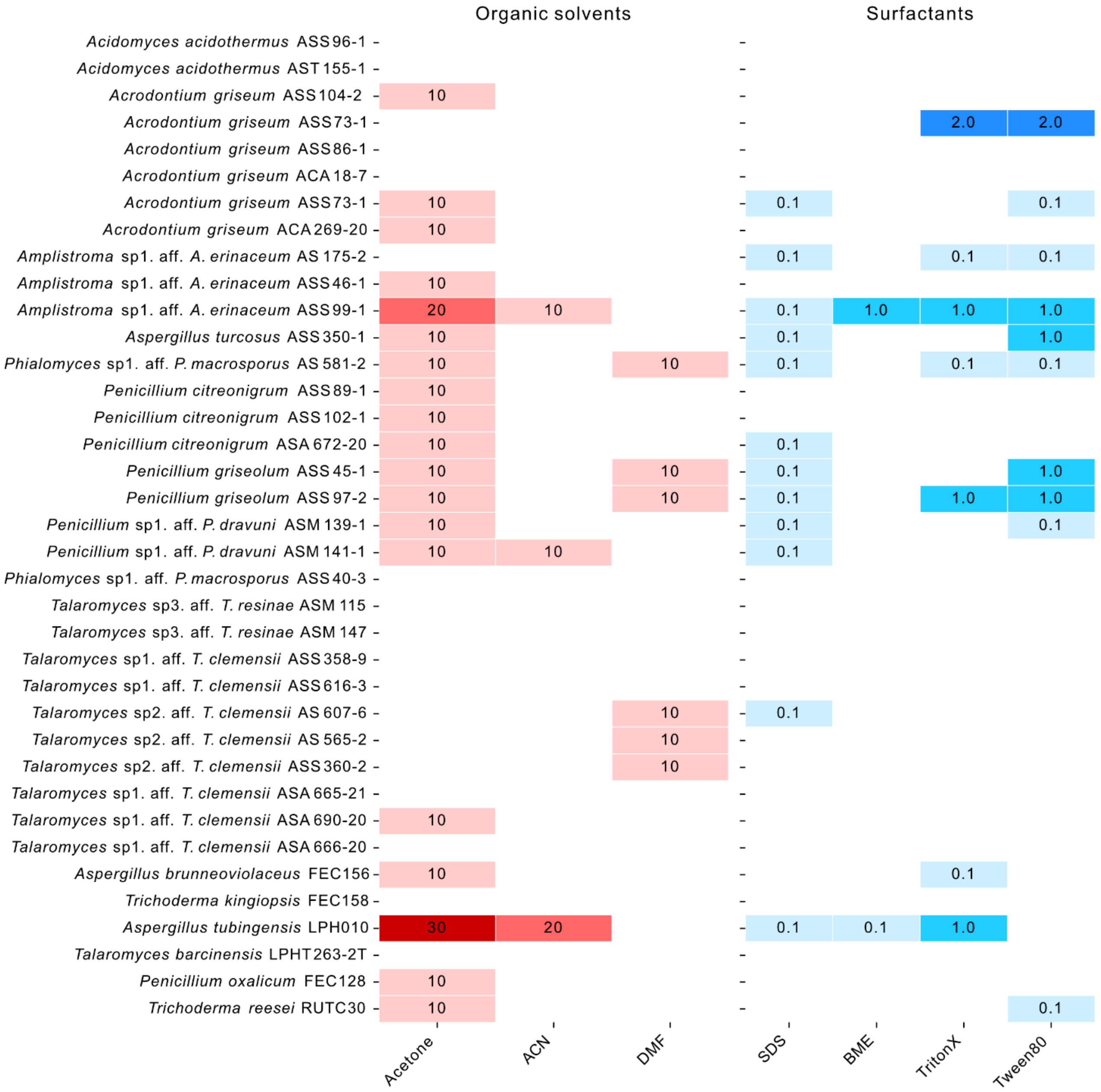

Figure S3. Resistance of fungal secretomes to organic solvents and surfactants. “Resistance” here is defined as the maximum concentration (% v/v) of each organic solvent or surfactant that could be used before xylanase activity (U/mL) decreased to <50% of the control activity (0% added chemical). Empty cells indicate that the activity fell to <50% of the control activity at the lowest tested solvent or surfactant concentration. Organic solvents tested were acetone, acetonitrile (ACN), and dimethylformamide (DMF; each tested: 10, 20, and 30%); surfactants used were sodium dodecyl sulfate (SDS), β-mercaptoethanol (BME), Triton X, and Tween 80 (each tested at 0.1, 1, and 2%).

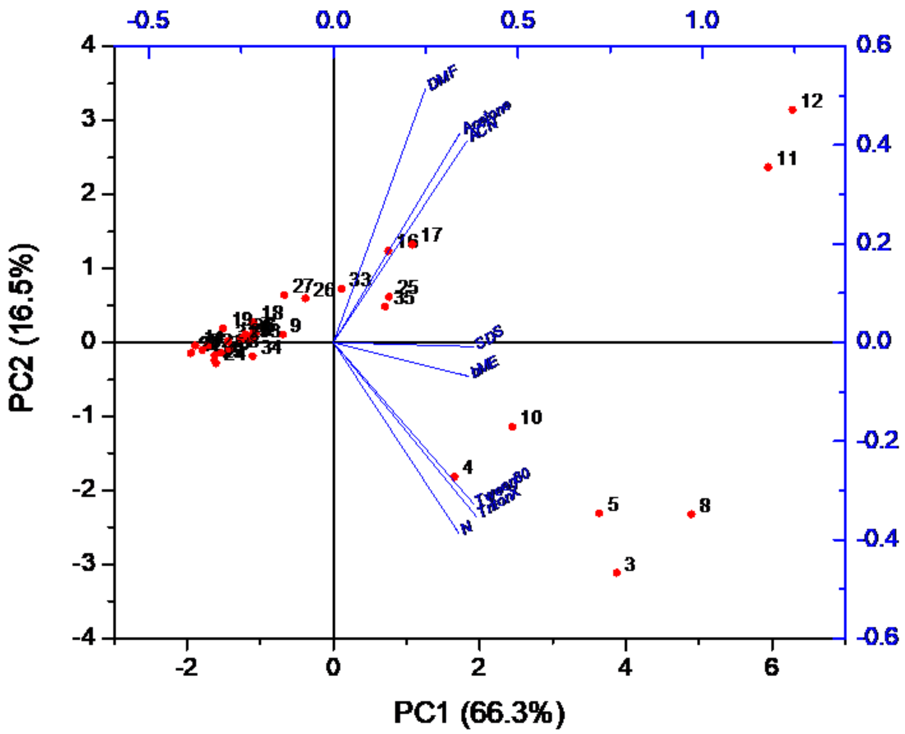

Figure S4. Principle Component Analysis of organic solvents and surfactants. Red dots: Each species' scores. Blue: Loading level vectors of each organic solvent/surfactant on PC1 and 2. The abbreviations are the same as Figure S3. "N" denotes the no solvent condition.

Table S1. Summary of taxa that are currently classified as acidophilic and acidotolerant filamentous fungi.

Species	Mycobank ID	Synonym/Original name	Phylum	Source of isolation	Lowest growth pH	Country	Genome sequence available	Reference
Acidea extrema	MB#805425	*Acidea extrema*	Ascomycota	Highly acidic soil	2.0 (medium)	Czechia	nd	(Hujslová et al. 2014)
Acidiella bohemica	MB#564519	*Acidiella bohemica*	Ascomycota	Extremely acidic soil	2.0 (medium)	Czechia	**Yes**	(Hujslová et al. 2012)
		Fodinomyces uranophilus =Acidiella uranophila	Ascomycota	Process waters (raffinate) from uranium mine	2.0 (medium)	Australia	nd	(Vázquez-Campos et al. 2014)
Acidiella polonica	MB#833242	*Acidiella polonica*	Ascomycota	Acidic sulphide-rich technosol	1.0 (medium)	Poland	nd	(Kolařík et al. 2021)
Acidomyces acidophilus	MB#511856	Fungus D	Ascomycota	Industrial solution containing 4% copper sulfate	0.2–0.7 (environment)	United States	nd	(Starkey and Waksman 1943)
		Scytalidium acidophilium	Ascomycota	Soil near a sulphur pile at a gas purification plant	1.4-3.5 (environment)	Canada	nd	(Sigler and Carmichael 1974)
		Bispora sp. MEY-1	Ascomycota	Acidic wastewater of a uranium mine	2.5-3.0 (media, optimum)	China	nd	(Luo et al. 2009)
Acidomyces acidothermus	MB#804969	*Acidomyces richmondensis*	Ascomycota	Acid mine drainage	0.8–1.38 (environment)	United States	**Yes**	(Baker et al. 2004)
		Teratosphaeria acidotherma	**Ascomycota (*)**	Biofilms from acidic hot spring	1.0 (medium)	Japan	nd	(Yamazaki et al. 2010)
Acidothrix acidophila	MB#805424	*Acidothrix acidophila*	Ascomycota	Highly acidic soil	1.0 (medium)	Czechia	nd	(Hujslová et al. 2014)
Acontium velatum	MB#142596	*Acontium velatum*	Ascomycota	Industrial solution containing 4% copper sulfate	0.2–0.7 (environment)	United States	nd	(Starkey and Waksman 1943)
Coniochaeta fodinicola	MB#807117	*Coniochaeta fodinicola*	Ascomycota	Process waters (raffinate) from uranium mine	1.0 (medium)	Australia	nd	(Vázquez-Campos et al. 2014)
Neohortaea acidophila	MB#807810	*Hortaea acidophila*	Ascomycota	Brown coal laboratory extract	0.6 (environment)	Germany	**Yes**	(Hölker et al. 2004)
Penicillium corylophilum	MB#178294	*Penicillium corylophilum*	Ascomycota	Acidic water, copper recovery plant	1.0 (medium)	United States	**Yes**	(Sinclair and Herring 1975)
Tardiomyces digboiensis	MB#853436	*Candida digboiensis*	Ascomycota	Acidic tar sludge-contaminated oil field	2.0 (environment)	India	**Yes**	(Prasad et al. 2005)
Goffeauzyma aciditolerans	MB#813252	*Cryptococcus aciditolerans*	**Basidiomycota**	Pyrite mine drainage	2.2 (environment)	Portugal	nd	(Gadanho and Sampaio 2009)
Goffeauzyma iberica	MB#813256	*Cryptococcus ibericus*	**Basidiomycota**	Pyrite mine drainage	2.2 (environment)	Portugal	nd	(Gadanho and Sampaio 2009)
Goffeauzyma metallitolerans	MB#813257	*Cryptococcus metallitolerans*	**Basidiomycota**	Pyrite mine drainage	2.2 (environment)	Portugal	nd	(Gadanho and Sampaio 2009)
Takashimella tepidaria	MB#810687	*Cryptococcus tepidarius*	**Basidiomycota**	A stream from a hot spring	1.2 (medium)	Japan	**Yes**	(Takashima et al. 2009)

(*): The sexual state of *Teratosphaeria acidotherma* was Teleomorph. All others shown here were in the Anamorphic state. **nd:** no data.

Table S2. PCA loading coefficients for each organic solvent and surfactant.

	PC1	PC2
No solvent	0.340	-0.388
Acetone 10%	0.343	0.424
ACN 10%	0.362	0.409
DMF 10%	0.25	0.516
SDS 0.1%	0.381	-0.00860
βME 0.1%	0.366	-0.0687
Triton X 0.1%	0.386	-0.353
Tween 80 0.1%	0.381	-0.329

Supplementary references

Baker, B. J.; Lutz, M. A.; Dawson, S. C.; Bond, P. L.; Banfield, J. F., Metabolically active eukaryotic communities in extremely acidic mine drainage. *Applied and Environmental Microbiology* 2004, *70* (10), pp 6264-6271.

Gadanho, M.; Sampaio, J. P., *Cryptococcus ibericus* sp. nov., *Cryptococcus aciditolerans* sp. nov. and *Cryptococcus metallitolerans* sp. nov., a new ecoclade of anamorphic basidiomycetous yeast species from an extreme environment associated with acid rock drainage in São Domingos pyrite mine, Portugal. *International Journal of Systematic and Evolutionary Microbiology* 2009, *59* (9), pp 2375-2379.

Hölker, U.; Bend, J.; Pracht, R.; Tetsch, L.; Müller, T.; Höfer, M.; Hoog, G. S., *Hortaea acidophila,* a new acid-tolerant black yeast from lignite. *Antonie Van Leeuwenhoek* 2004, *86* (4), pp 287-294.

Hujslová, M.; Kubátová, A.; Kostovčík, M.; Blanchette, R. A.; de Beer, Z. W.; Chudíčková, M.; Kolařík, M., Three new genera of fungi from extremely acidic soils. *Mycological Progress* 2014, *13* (3), pp 819-831.

Hujslová, M.; Kubátová, A.; Kostovcík, M.; Kolarík, M., *Acidiella bohemica* gen. et. sp. nov. and *Acidomyces* spp. (Teratosphaeriaceae), the indigenous inhabitants of extremely acidic soils in Europe. *Fungal Diversity* 2012, *58* (1), pp 33-45.

Kolařík, M.; Stępniewska, H.; Jankowiak, R., Taxonomic revision of the acidophilic genus *Acidiella* (Dothideomycetes, Capnodiales) with a description of new species from Poland. *Plant Systematics and Evolution* 2021, *307* (3), p 38.

Luo, H.; Wang, Y.; Wang, H.; Yang, J.; Yang, Y.; Huang, H.; Yang, P.; Bai, Y.; Shi, P.; Fan, Y.; Yao, B., A novel highly acidic β-mannanase from the acidophilic fungus *Bispora* sp. MEY-1: gene cloning and overexpression in *Pichia pastoris*. *Applied Microbiology and Biotechnology* 2009, *82* (3), pp 453-461.

Prasad, G. S.; Mayilraj, S.; Sood, N.; Singh, V.; Biswas, K.; Lal, B., *Candida digboiensis* sp. nov., a novel anamorphic yeast species from an acidic tar sludge-contaminated oilfield. *International Journal of Systematic and Evolutionary Microbiology* 2005, *55* (2), pp 967-972.

Sigler, L.; Carmichael, J. W., A new acidophilic *Scytalidium*. *Canadian Journal of Microbiology* 1974, *20* (2), pp 267-8.

Sinclair, N. A.; Herring, C. M., Isolation of *Penicillium corylophium* Dierckx from acid mine water and its optimal growth on hydrocarbons at acid pH. *Mycopathologia* 1975, *57* (1), pp 19-22.

Starkey, R. L.; Waksman, S. A., Fungi tolerant to extreme acidity and high concentrations of copper sulfate. *Journal of Bacteriology* 1943, *45* (5), pp 509-19.

Takashima, M.; Sugita, T.; Toriumi, Y.; Nakase, T., *Cryptococcus tepidarius* sp. nov., a thermotolerant yeast species isolated from a stream from a hot-spring area in Japan. *International Journal of Systematic and Evolutionary Microbiology* 2009, *59* (1), pp 181-185.

Vázquez-Campos, X.; Kinsela, A. S.; Waite, T. D.; Collins, R. N.; Neilan, B. A., *Fodinomyces uranophilus* gen. nov. sp. nov. and *Coniochaeta fodinicola* sp. nov., two uranium mine-inhabiting Ascomycota fungi from northern Australia. *Mycologia* 2014, *106* (6), pp 1073-1089.

Yamazaki, A.; Toyama, K.; Nakagiri, A., A new acidophilic fungus *Teratosphaeria acidotherma* (Capnodiales, Ascomycota) from a hot spring. *Mycoscience* 2010, *51* (6), pp 443-455.
